# Spatial dependency of Buruli ulcer prevalence on arsenic-enriched domains in Amansie West District, Ghana: implications for arsenic mediation in *Mycobacterium ulcerans *infection

**DOI:** 10.1186/1476-072X-3-19

**Published:** 2004-09-15

**Authors:** Alfred A Duker, Emmanuel JM Carranza, Martin Hale

**Affiliations:** 1 International Institute for Geo-information Science and Earth Observation (ITC), Enschede, P.O. Box 6, 7500 AA The Netherlands

## Abstract

**Background:**

In 1998, the World Health Organization recognized Buruli ulcer (BU), a human skin disease caused by *Mycobacterium ulcerans *(MU), as the third most prevalent mycobacterial disease. In Ghana, there have been more than 2000 reported cases in the last ten years; outbreaks have occurred in at least 90 of its 110 administrative districts. In one of the worst affected districts, Amansie West, there are arsenic-enriched surface environments resulting from the oxidation of arsenic-bearing minerals, occurring naturally in mineral deposits.

**Results:**

Proximity analysis, carried out to determine spatial relationships between BU-affected areas and arsenic-enriched farmlands and arsenic-enriched drainage channels in the Amansie West District, showed that mean BU prevalence in settlements along arsenic-enriched drainages and within arsenic-enriched farmlands is greater than elsewhere. Furthermore, mean BU prevalence is greater along arsenic-enriched drainages than within arsenic-enriched farmlands.

**Conclusion:**

The results suggest that arsenic in the environment may play a contributory role in MU infection.

## Background

Buruli ulcer (BU) is a skin disease, which usually begins as a painless nodule or papule and may progress to massive skin ulceration. If untreated BU may lead to extensive soft tissue destruction, with inflammation extending to deep fascia. The parts of the body most affected are the extremities. Subsequent complications may include contractural deformities. The main form of treatment is wide excisional surgery, including amputation of limbs, which requires prolonged hospitalization and is thus a significant burden on hospital resources and budgets.

In recent years, there has been increased incidence of BU in West Africa (including Benin, Burkina Faso, Cote d'Ivoire, Ghana, Guinea, Liberia and Togo), Mexico, French Guyana, Papua New Guinea and Australia. The disease seems to affect mostly impoverished inhabitants in remote and rural areas; children are the most vulnerable, accounting for about 70% of the cases [1]. The World Health Organization (WHO) has recognized BU as the third most prevalent mycobacterial disease after tuberculosis and leprosy and has called for urgent action to control it [2].

The causative agent of BU is *Mycobacterium ulcerans *(MU), which was first described in Bainsdale, Australia, in 1948 [3]. From the medical point of view, MU is among the group of mycobacteria that are potentially pathogenic in humans and animals under special circumstances [4]. It is suggested MU enters through a small break or trauma in the skin because it is not known to penetrate through intact or healthy skin [5,6]. Portaels et al. [7] have suggested that insects may be involved in the transmission of the disease because insects found in the roots of trees tested positive with the mycobacterium. Marsolliers et al. [8] found through an experimental study that the bite of MU-infected waterborne insects transmitted infection to mice. In terms of human infection, however, the reservoir of MU and the mode of transmission of BU are still unclear [4,9-12].

Epidemiological data suggest that environmental factors such as climate, soil, geology, geochemistry, etc. may indirectly influence or contribute to MU infection [4]. In addition, the frequencies of some diseases caused by mycobacteria indicate that species are distributed geographically [13]. For example, MU has been observed mainly in the tropics and [4] especially in anthropogenically-polluted areas [14].

Since MU is known to be present in nature although its reservoir is not known and since the epidemiology of BU is still unclear, there is a need to have a better understanding of environmental, ecological, and behavioural factors that predispose to infection. Spatial analysis potentially contributes important information leading to the understanding of the epidemiology and etiology of BU. The main objective of this paper is to explore relationships between some spatial environmental factors and the prevalence of BU.

## Results

**Figure 4:**
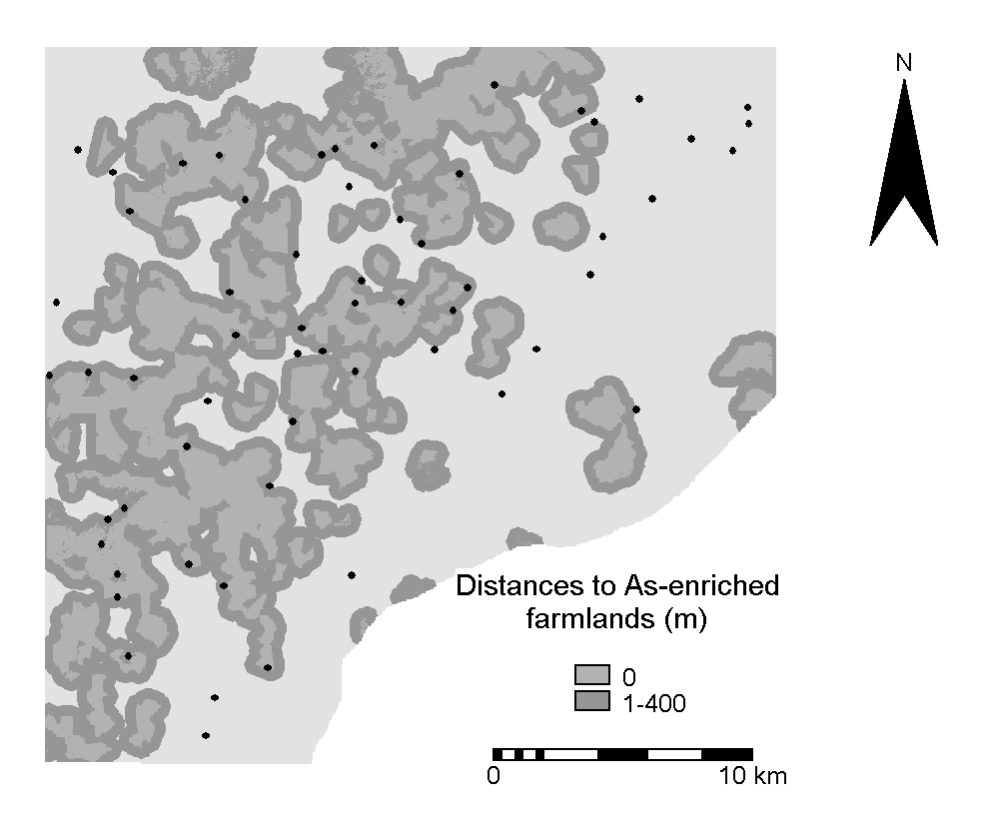
**Map of As-enriched farmlands. **Distances to As-enriched farmlands (red; with > 15 ppm As in stream sediments) and locations of villages with BU cases.

**Figure 5:**
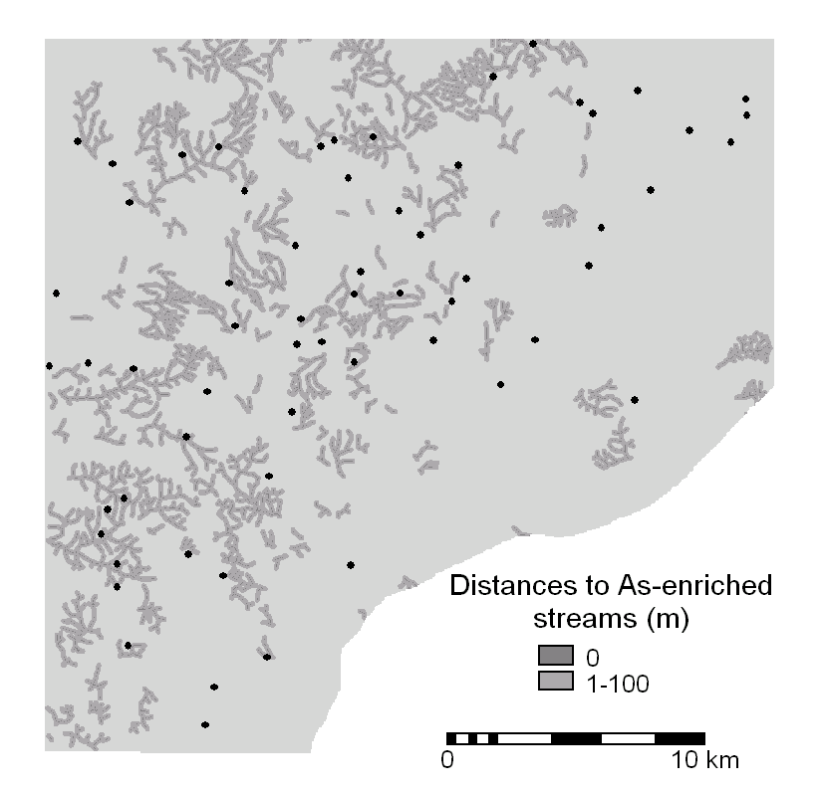
**Map of As-enriched streams. **Distances to As-enriched streams (black; with > 15 ppm As in stream sediments) and locations of villages with BU cases.

For the buffers tested, *P *values range from 0.09 to 0.46. The buffers with the highest *P *values (i.e., 0.09) are 100 m for drainage channels and 400 m for farmlands. With these buffers 24 of the 61 settlements (i.e., 39%) fall within 100 m of arsenic-enriched drainage channels (Figure 4) and 41 of the 61 settlements (i.e., 67%) fall within 400 m of As-enriched farmlands (Figure 5). The mean BU prevalence within the drainage buffer is 0.7% whereas the mean BU prevalence inside the farmland buffer is 0.6%. Thus the naturally smaller number of settlements within the drainage buffer (i.e., 24) has a slightly higher BU prevalence than the relatively larger number of settlements within the farmland buffer (i.e., 41).

## Discussion

Siting of rural settlements in the study area is based primarily on proximity to and availability of water for drinking and other domestic purposes. Consequently, many settlements are located within the optimum buffer distance of 100 m from drainage channels. Where water is abstracted from drainage channels enriched in arsenic, chronic ingestion of arsenic-enriched water through drinking and cooking is likely. This renders the inhabitants susceptible to several kinds of diseases [15,16] including BU. Amofah et al. [17] studied 90 BU patients and found that 52 used surface water as the source of their drinking water. The result of the statistical analysis corroborates this observation in that BU prevalence is highest where the inhabitants have ready access to domestic water supplies from arsenic-enriched surface drainage.

Subsistence farmlands, especially those that depend partially on irrigation, tend to be located along stream floodplains. Soils in these floodplains have a high cation exchange capacity [18] so that, where streams carry high concentration of arsenic, there is accumulation of arsenic in the soils of the floodplains. These high concentrations of arsenic are in part taken up by the foodcrops grown there [19-21]. The results of the statistical analysis suggest that a high proportion of settlements with high BU prevalence exploit such floodplain farmlands enriched in arsenic.

Through consumption of arsenic-enriched drinking water and arsenic-enriched foodcrops, inhabitants in some settlements in the Amansie West District are prone to chronic ingestion of higher-than-average (but sub-toxic) levels of arsenic. Arsenic interacts with and inhibits several enzymes in the body [15] leading to several multisystemic non-cancer effects [16], which could predispose to defect the immune system [22]. Subjects exposed to high levels of arsenic concentrations were known to have impaired immune response [23]. Immunosuppression due to arsenic has been found to defect antigen processing of splenic macrophages with consequent defective mechanism of helper T-cells [24,25]. Down-regulation of the immune system is known to be a risk factor for the development of BU [26,27]. Several studies [e.g., [28-31]] have reported of impaired resistance to viral/bacterial infection via arsenic ingestion.

## Conclusions

The results of this study reveal spatial dependency of BU prevalence upon proximity to drainage channels and farmlands containing > 15 ppm arsenic. Proximity implies chronic exposure to and/or ingestion of elevated concentrations of arsenic, which influences susceptibility to infection.

## Methods

### Research hypotheses

It has been consistently theorized that BU is acquired when MU enters the body through a skin rupture [32,27]. However, several people who were affected by the disease do not recall having any break or trauma in their skin prior to being infected [33]. A possible alternative is entry through non-ruptured but unusually unhealthy or thin skin.

Several dermatological diseases (e.g., Bowen's disease, hyperkeratosis, hyperpigmentation) are related to arsenic ingestion and exposure [34]. Bioaccumulation of arsenic in the fatty tissues of the skin [35], due to its high lipid solubility [36,37], may provide a favourable environment for MU in the skin because arsenic is known to help microorganisms grow [38]. It can be hypothesized, therefore, that (a) arsenic induces MU adhesion to human tissues and (b) arsenic influences the ability of MU to establish BU.

In a case study, Amofah et al. [17] reported that about 44% of the BU patients were farmers whilst about 54% were school children. In Ghana many children help their parents on farms. Not only do farmers and children come in contact with natural drainage areas on their journeys to and from their farmlands, but also the farms are located near water bodies or drainage systems for obvious irrigation purposes [39]. If farmlands and surface drainage channels are contributory factors to BU, farmlands and surface drainage channels enriched in arsenic may contribute to still higher prevalence of BU.

### Research methodology

Spatial analysis of data provides opportunities for epidemiologists to study associations between environmental factors and spatial distribution of diseases [40]. A geographic information system (GIS) is capable of analyzing and integrating large quantities of geographically distributed data as well as linking geographic data to non-geographic data to generate information useful in further scientific (or medical) research and in decision-making.

In this study, topographic map data, stream sediment geochemical data for arsenic, ASTER satellite imagery and locations of settlements with BU cases were the basic data inputs into the GIS. Spatial data processing was carried out (a) to delineate arsenic-enriched catchment basins based on arsenic concentrations in stream sediment samples, (b) to delineate farmlands from ASTER satellite imagery and determine arsenic-enriched farmlands based on catchment basin data and (c) to extract drainage channels from the topographic map and determine arsenic-enriched drainage channels based on arsenic-enriched catchment basins. Proximity analysis was undertaken to determine spatial relationships between BU-affected areas and the arsenic-enriched areas determined from the data inputs.

### The study area

#### History of BU in Ghana

**Figure 1:**
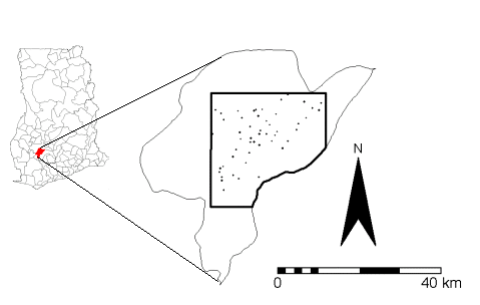
**The study area. **Amansie West District, Ghana, showing the study area (box) and villages with BU cases (black dots).

The study area is in Ghana, where the first case of BU was reported in 1971 and, between 1991 and 1997, more than 2000 cases have been reported [41]. The disease has affected all of the ten regions and at least 90 of the 110 districts in Ghana [42]. The Ashanti Region is the worst affected, accounting for about 60% of all reported cases, of which the greatest percentage is in the Amansie West District (Figure 1).

#### Location of study area

The Amansie West District lies between latitudes 6°N and 6°45'N and longitudes 1°30'W and 2°15'W. It covers an area of about 1,136 km^2^. The district capital, Manso Nkwanta, is about 40 km south of Kumasi. The district is drained by the Offin and Oda rivers. Vegetation in the district is composed mainly of secondary forests, thicket, forb regrowth (i.e., soft-stemmed leafy herbs, mostly the weeds, which appear on farms and have to be cut regularly) and swamp vegetation. Vegetation thrives in ferric fluvisols, which are the major soil types in the district. These soils have been developed through yearly rainfall ranging from 125 to 200 cm with temperatures of 22 to 30°C. The landscape of the district varies from gentle to broken.

The district is underlain by Lower Proterozoic Birimian and, to a lesser extent, Tarkwaian rocks. Throughout Ghana, Birimian rocks of West Africa are mainly volcanic greenstones with intervening sedimentary rocks and granitoid intrusions, in places containing deposits composed of pyrite, arsenopyrite, minor chalcopyrite, sphalerite, galena, native gold and secondary hematite [43].

The district has about 310 settlements (though not all these settlements are mapped) with a population in 2000 of 108,726. There are approximately equal percentages of males and females (49% and 51%, respectively), of whom 70% are farmers and 22% are engaged in legal and 'galamsey' (or illegal) mining.

The study area is the east-central part of the Amansie West District (covered by a single topographic map sheet, 0602C1), with an area of about 623 km^2^, comprising 61 settlements and including the Bilpraw goldmine (formerly a treasure mine of the Ashanti Kings). The BU cases per settlement range from 1–29.

#### Materials

The following are the sources of spatial data input to the GIS.

• Incidence of BU per settlement in 1999, obtained from Korle-BU Teaching Hospital, Accra, Ghana.

• Settlement population estimates for 2000, projected by the Ministry of local government and rural development.

• Topographic map (Sheet 0602C1, 1974, at a scale of 1: 50,000), a single sheet covering the study area, obtained from the Survey Department, Accra, Ghana.

• Location map (at scale of 1: 62,500) of stream sediment samples collected in part of the Amansie West District in 1992 and list of arsenic concentrations determined in these samples, obtained from the Geological Survey Department, Accra, Ghana.

• Boundary map (at scale of 1: 250,000, surveyed in 1991) of the district, obtained from the Amansie West District Administration.

• ASTER imagery (level 1B) acquired on 15/01/2002, obtained from the US Geological Survey.

• Landuse/landcover map of Ghana (traced on Landsat TM data of 1998 and published in the same year), obtained from the Remote Sensing Application Unit (RSAU), University of Ghana, Legon.

The GIS operations were carried out in three principal steps: (1) spatial data capture; (2) generation of spatial factor maps; and (c) spatial data analysis. The GIS operations were carried out using ILWIS (Integrated Land and Water Information Systems), a GIS software package developed by the International Institute for Geo-information Science and Earth Observation (ITC) in the Netherlands.

#### Spatial data capture

The different analog maps were scanned then georeferenced (by defining the x and y coordinates of the corner points of the maps) into a UTM coordinate system. From the scanned map, spatial data were captured by screen digitizing. From the topographic map, rivers, streams and gullies were digitised as line segments as were elevation contours. The boundaries of the district were digitised as line segments and then polygonized. The locations of centres of 61 settlements (identifiable on the topographic map) were digitised as points and the BU incidence in 1999 was recorded as spatial attribute of each settlement.

From the stream sediment sample location map, the locations of the samples were digitised as points and the arsenic concentrations (in ppm) were recorded as a spatial attribute of each sample. The ASTER imagery was also georeferenced to the same coordinate system using eight reference points (tie points), which were selected in the image and which could be identified in the topographic map. Using an affine transformation, a root mean square error (RMSE) of 0.58 pixel was obtained in georeferencing the ASTER imagery.

For each of the settlements with incidence of BU the percentage prevalence of BU was calculated. Prevalence expresses cases of a disease in terms of the proportion of the population afflicted at a specified time [44]. It is expressed here as the number of BU cases in a settlement in 1999 divided by the estimated population in 2000 multiplied by 100 to yield a percentage.

#### Spatial factor maps

The spatial factor maps generated from the stream sediment geochemistry data for use in the spatial analysis were: (a) map of arsenic-enriched catchment basins; (b) map of arsenic-enriched farmlands; (c) map of arsenic-enriched drainage channels.

##### Arsenic-enriched catchment basins

The stream sediment geochemical data for arsenic were initially analysed statistically to determine a threshold value that divides the data into background (normal) classes and anomalous (abnormally high) classes of arsenic concentrations. The data are lognormally distributed and, after removing obvious outliers in the data, a geometric mean of 8.9 ppm As and standard deviation of 2.8 ppm As were obtained. The threshold value was therefore set at 15 ppm As (i.e., approximately the mean plus two standard deviations). The spatial distribution of arsenic was then mapped through the generation of a catchment basin anomaly map in which a sample catchment basin is assigned the geochemical attribute of the corresponding sample [45,46]. Generation of sample catchment basins involved the following steps (using ILWIS):

• creation of a raster digital elevation model (DEM) through interpolation of elevation contours;

• generation of raster map of drainage lines; and

• calculation of sample catchment basin boundaries via an iterative calculation procedure involving the DEM and the raster map of drainage lines.

**Figure 2:**
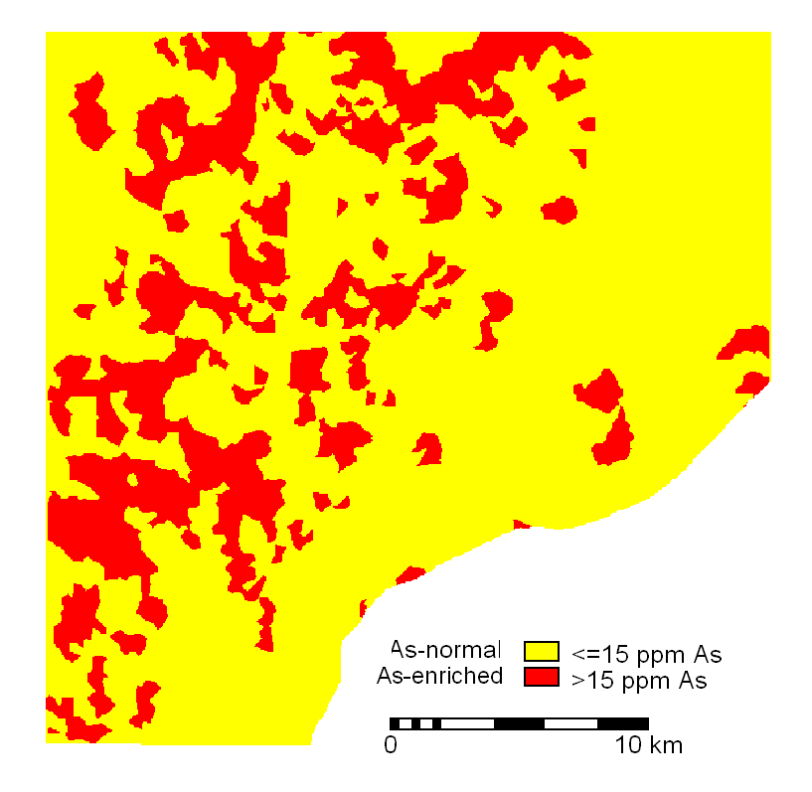
**Binary map of the catchment basin. **Binary map (of the catchment basin) showing arsenic-normal and arsenic-enriched areas.

The catchment basin map of arsenic concentrations was then classified into a binary map showing arsenic-normal areas (with ≤ 15 ppm As) and arsenic-enriched areas (with > 15 ppm As) as shown in Figure 2. About 24% of the study area is occupied by arsenic-enriched catchment basins.

##### Arsenic-enriched farmlands

**Figure 3:**
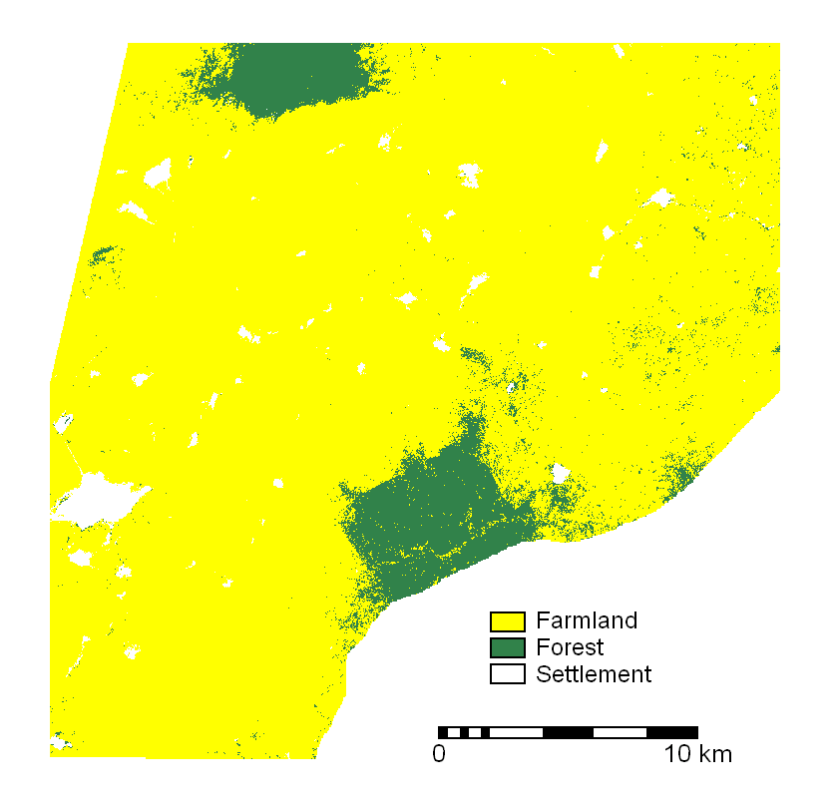
**Landcover/landuse map. **Landcover/landuse map based on supervised classification of ASTER data.

A supervised classification of ASTER imagery was carried out to distinguish between the major landcover/landuse classes known in the area. These landcover classes are (a) forest areas, (b) residential areas or settlements (bare of vegetation), and (c) farmlands. Using the available landuse/landcover map and topographic map as references, training pixels of known landuse/landcover classes were selected using a colour composite of ASTER bands 2, 3 and 4. These three bands gave the highest optimal index factor (OIF), which indicates the combination of three spectral bands that provide optimum information about landcover [47]. The box classifier [48] was chosen for the image classification. The classified image (Figure 3), which was also validated in the field, has an overall accuracy of at least 91% with reference to the landcover/landuse map. The classified image indicates that about 91% of the area is farmland.

To determine arsenic-enriched farmlands, a Boolean AND operation was performed by crossing the catchment basin anomaly map and the classified landcover/landuse image. About 21% of the total area of farmlands in the classified image is arsenic-enriched.

##### Arsenic-enriched portions of the drainage systems

A Boolean AND operation was performed by crossing the catchment basin anomaly map and the raster map of drainage lines. About 22% of the total length of drainage lines is indicated to be arsenic-enriched.

#### Spatial data analysis

The inhabitants of a settlement earn their livelihoods by exploiting the resources of the surrounding land. This land influences their exposure to infections and to environmental factors that dispose to infections. Proximity analysis was therefore used to determine spatial relationships between BU prevalence per settlement and (i) arsenic-enriched farmlands and (ii) arsenic-enriched portions of the drainage system. The proximity analysis was carried in two principal steps. First, maps of distances from arsenic-enriched farmlands and arsenic-enriched portions of the drainage system were generated. Second, the point map of BU prevalence per settlement was overlaid on (or crossed with) each of these maps.

A buffer is a zone of specified distance around a selected map feature. A GIS creates buffer zones around selected map features such as arsenic-enriched farmlands and arsenic-enriched portions of drainage systems. Around each of these, buffers were set at intervals of 100 m up to 1000 m. Each buffer zone map was crossed with BU prevalence data of settlement to determine how many of these fall within and outside of the buffer.

At each increasing interval of 100 m, a test of the significance of the difference of the mean BU prevalence within the buffer and outside of the buffer is made using the t-statistic:



where ,  are the sample means,  is the pooled sample variance, *n_*i *_*and *n_*j *_*are the sample sizes from population *i *and *j*. Using *t_*ij *_*and degrees of freedom given by *n_*i *_*+ *n_*j*_*-2, a *t *distribution look-up table provides the probability, *P *that the means are significantly different.

## Authors' contributions

AAD carried out the research and drafted the manuscript. EJMC guided parts of the research and both EJMC and MH reviewed the manuscript.
